# Retrospective batch analysis to evaluate the diagnostic accuracy of a clinically deployed AI algorithm for the detection of acute pulmonary embolism on CTPA

**DOI:** 10.1186/s13244-023-01454-1

**Published:** 2023-06-06

**Authors:** Eline Langius-Wiffen, Pim A. de Jong, Firdaus A. Mohamed Hoesein, Lisette Dekker, Andor F. van den Hoven, Ingrid M. Nijholt, Martijn F. Boomsma, Wouter B. Veldhuis

**Affiliations:** 1 Department of Radiology, Isala Hospital, Dr. van Heesweg 2, 8025 AB Zwolle, The Netherlands; 2 Department of Radiology, University Medical Centre Utrecht, Utrecht, The Netherlands; 3 Department of Nuclear Medicine, St. Antonius Hospital, Nieuwegein, The Netherlands; 4 Division of Imaging and Oncology, University Medical Centre Utrecht, Utrecht, The Netherlands

**Keywords:** Artificial intelligence, Pulmonary embolism, Computed tomography angiography, Retrospective studies

## Abstract

**Purpose:**

To generate and extend the evidence on the clinical validity of an artificial intelligence (AI) algorithm to detect acute pulmonary embolism (PE) on CT pulmonary angiography (CTPA) of patients suspected of PE and to evaluate the possibility of reducing the risk of missed findings in clinical practice with AI-assisted reporting.

**Methods:**

Consecutive CTPA scan data of 3316 patients referred because of suspected PE between 24-2-2018 and 31-12-2020 were retrospectively analysed by a CE-certified and FDA-approved AI algorithm. The output of the AI was compared with the attending radiologists’ report. To define the reference standard, discordant findings were independently evaluated by two readers. In case of disagreement, an experienced cardiothoracic radiologist adjudicated.

**Results:**

According to the reference standard, PE was present in 717 patients (21.6%). PE was missed by the AI in 23 patients, while the attending radiologist missed 60 PE. The AI detected 2 false positives and the attending radiologist 9. The sensitivity for the detection of PE by the AI algorithm was significantly higher compared to the radiology report (96.8% vs. 91.6%, *p* < 0.001). Specificity of the AI was also significantly higher (99.9% vs. 99.7%, *p* = 0.035). NPV and PPV of the AI were also significantly higher than the radiology report.

**Conclusion:**

The AI algorithm showed a significantly higher diagnostic accuracy for the detection of PE on CTPA compared to the report of the attending radiologist. This finding indicates that missed positive findings could be prevented with the implementation of AI-assisted reporting in daily clinical practice.

**Critical relevance statement:**

Missed positive findings on CTPA of patients suspected of pulmonary embolism can be prevented with the implementation of AI-assisted care.

**Key points:**

- The AI algorithm showed excellent diagnostic accuracy detecting PE on CTPA.
- Accuracy of the AI was significantly higher compared to the attending radiologist.
- Highest diagnostic accuracy can likely be achieved by radiologists supported by AI.
- Our results indicate that implementation of AI-assisted reporting could reduce the number of missed positive findings.

**Graphical abstract:**

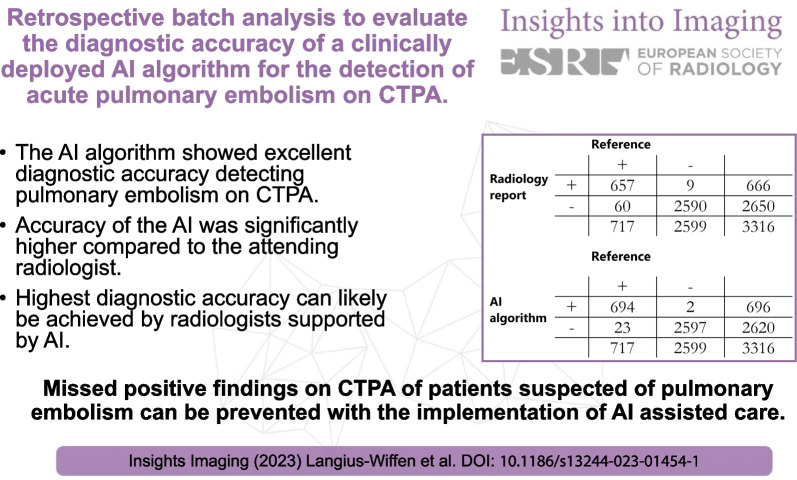

## Background

Workload for radiologists during regular working hours and on-call hours has dramatically increased in the last decades. CT scans during on-call hours were reported to have increased with 500% between 2006 and 2020 and the number of CT pulmonary angiography (CTPA) to detect pulmonary embolism (PE) even showed an increase of 1360% [1]. As many studies continue to show the added value of medical imaging in patient care and technical developments lead to the acquisition of more complex and larger datasets, the workload is expected to increase even further in the future [2].

Unfortunately, the increased workload is not without risk to the quality of radiologists’ reports, as it has been shown to result in radiologists developing reading fatigue, which may cause diagnostic errors [3, 4], and additionally in an increased rate of burn-out among radiologists and residents [5]. Missed PE can influence patient care and outcome, as it is a potentially life threatening condition with risk of developing severe complications such as pulmonary hypertension. Radiologists reading CTPA have shown excellent performance to detect PE [6, 7], but the continued increase in workload could lead to additional missed findings in the future.

Artificial intelligence (AI) applications are increasingly evaluated in studies to determine their value in supporting radiologists through workflow improvement and assisted diagnosis [8].

Various requirements have to be met in order to guarantee successful clinical implementation of AI in daily practice [9, 10]. Besides technical validity, clinical validity needs to be assessed in diagnostic accuracy studies. A limited number of studies were already reported on the diagnostic performance of AI algorithms to detect PE on CTPA [11–16]. A recent meta-analysis reviewed five studies and showed variability in performance of the different AI algorithms in detecting PE in research settings [17]. Possible reasons for the discrepancy in results could be that not all of the algorithms had been thoroughly externally validated for use in clinical practice and/or the large variation in sample sizes and PE prevalence [18]. A commercially available FDA-approved and CE-marked AI algorithm to detect PE on CTPA has shown a promising diagnostic accuracy to detect PE with a sensitivity and specificity of over 90% [12, 15]. Cheikh et al. showed that this AI detected 19 PE that were missed by the initial report in a sample of 1202 CTPA with a PE prevalence of 15,8%, resulting in a higher sensitivity of the AI than the initial report. However, the AI had a significantly lower specificity leading to more false positives. Thus, the AI could cause overdiagnosis when radiologists would rely on the algorithm, which raises the question whether widespread implementation is currently beneficial and safe [9].

The goal of this comparative diagnostic accuracy study was to generate and extend the evidence on the performance of this FDA-approved and CE-marked AI algorithm to detect PE on a large sample of > 3000 CTPA and to determine the additional value of using AI assistance in daily clinical practice.

## Methods

### Patient inclusion

This comparative diagnostic accuracy study was approved by the institutional review board, and informed consent was waived (no. 2021-78371). CTPA scans of all consecutive patients ≥ 18 years referred to the Radiology department between 24-2-2018 and 31-12-2020 because of suspected PE were retrospectively included.

### Artificial intelligence algorithm

All anonymised scans were analysed by an FDA-approved and CE-marked PE (dedicated CTPA) AI algorithm (Aidoc Medical). The architecture of the algorithm has been recently described in detail by Petry et al. [19]. The algorithm was deployed on a virtual machine and integrated with our PACS (SECTRA). The CTPAs within our set time-frame were automatically analysed by the algorithm, where after we received AI activation maps that provided possible PE findings. Aidoc Medical was not involved in study design or analysis of the AI output they provided. No financial support was provided for this study. Their algorithm has not been trained on data originating from our hospital.

### Local reading and CT scanning protocol

In our hospital, CT scans are reviewed and reported just once by either a radiologist or by a radiology resident with an Entrusted Professional Activity level 3, 4 or 5 to read CTPA. During the study period, there was no algorithmic support for PE detection in usage.

The hospital had access to the Somaton Force (Siemens Healtineers), Brilliance 64 (Philips Healthcare), iCT (Philips Healthcare) and the IQon (Philips Healthcare). All CT scans were acquired and reconstructed with thin slices of about 1 mm.

The standard scanning protocol for CTPA included a bolus tracking in the pulmonary trunk with a trigger at 200 Hounsfield Units. The post threshold delay was 5 s (or minimal possible). The patients were instructed to maintain a regular breathing pattern and avoid deep inspiration. We used a flow of 5 mL/sec and inject 55–65 mL of contrast depending on body weight and 50 mL of NaCL, except for the spectral detector CT (IQon) where we used a kVp as low as possible to boost the iodine.

### Reference standard

The reference standard was established using the report by the attending radiologist or resident with adequate Entrusted Professional Activity level, the AI output and an evaluation of discordant cases. Cases classified as PE positive by both the radiology report and the AI were considered positive according to the reference standard. Similarly, cases classified as PE negative by both the radiology report and the AI were considered negative. Discordant cases were independently reviewed by a chest radiologist (P.A.J., > 10 years of experience) and a medical doctor (L.A.) who had access to the images, the radiology report and the AI output. When these readers agreed on the presence or absence of PE, the reference standard was set. In case of discrepancies a third adjudicator (a cardiothoracic radiologist (F.M.H., > 10 years of experience)) was consulted, who was given access to all available information, to determine the reference standard.

### Statistical analysis

For data analysis R version 4.2.0 was used (R Foundation for Statistical Computing, Vienna, Austria). All tests were two-sided. A *p* value < 0.05 was considered statistically significant.

Diagnostic accuracy measures of the AI algorithm were compared with the diagnostic accuracy measures of the radiology report using the DTComPair package of R. McNemar test was used to compare sensitivity and specificity. Relative predictive values were used to compare positive and negative predictive values.

## Results

### Patient inclusion

We included CT scans of 3316 patients suspected of PE (1615 females, 48.7%). The mean ± SD age was 58.6 ± 16.1 years. Given the retrospective nature of our study and the absence of informed consent, we did not have other clinical variables available in this study. All images were analysed by the AI.

### Radiology report versus AI output

In 633 patients, both the radiology report and the AI output were positive for PE. In 2588 patients, both the radiology report and the AI output were negative for PE. In 95 patients, the diagnosis was discrepant.

According to the reference standard, after re-evaluation by two readers and if needed adjudication, 717 CT patients were positive for PE, resulting in a prevalence of 21.6% (95% confidence interval 20.2–23.1%). Of those, 60 (8.4%) cases of PE were not reported by the attending radiologist and 23 (3.2%) were not detected by the AI algorithm. The cases of missed PE by the attending radiologist concerned two central/lobar, 12 segmental and 46 subsegmental PE. Solely peripheral PE were missed by the AI algorithm (7 segmental, 16 subsegmental). The attending radiologist reported 9 false positive findings, while the algorithm marked 2 false positives.

Overall, the algorithm showed significantly higher diagnostic accuracy measures compared to the radiology reports with sensitivity of 96.8% versus 91.6%, respectively, and specificity of 99.9% versus 99.7%. PPV and NPV of the AI algorithm were also significantly higher than of the radiology report (Table 1).

**Table 1:** Diagnostic accuracy measures of radiology report and AI algorithm on CTPA

	Radiology report	AI algorithm	*p* value
Sensitivity in % (95% CI)	91.6 (89.6–93.7)	96.8 (95.5–98.1)	*p* < 0.001
Specificity in % (95% CI)	99.7 (99.4–99.9)	99.9 (99.8–100.0)	*p* = 0.035
PPV in % (95% CI)	98.6 (97.8–99.5)	99.7 (99.3–100.0)	*p* = 0.030
NPV in % (95% CI)	97.8 (97.2–98.3)	99.1 (98.8–99.5)	*p* < 0.001

PPV = positive predictive value, NPV = negative predictive value, CI = confidence interval, *p* values concern the comparison between the diagnostic measures of the radiology report versus the AI algorithm

## Discussion

Our study showed that both radiologists and the FDA-approved and CE-marked AI algorithm have an excellent performance on CTPA, with a significantly higher diagnostic accuracy for the AI algorithm to detect PE on a large sample of CTPA of patients suspected of PE compared to the radiology report.

### Diagnostic accuracy AI algorithm

Only two previous studies reported on the performance of the FDA-approved and CE-marked automated integrated workflow AI algorithm to detect PE on CTPA used in our study. Weikert et al. reported that this AI algorithm had a sensitivity of 92.7% and specificity of 95.5% when analysing a sample of 1465 consecutive patients suspected of PE with a PE prevalence of 18.5% [12]. The initial report, reviewed by two physicians served as reference standard. Contrary to our study, they could not compare the performance of the AI to the initial report. A similar approach to ours was used by Cheikh et al., who compared the performance of the same AI algorithm to the initial report of the radiologist using a sample of 1202 consecutive patients suspected of PE from 3 different hospitals with a PE prevalence of 15.8%. They found that the sensitivity of the AI was slightly higher than the radiology report, albeit not significantly (92.6% versus 90%). However, radiologists showed a significantly higher specificity compared to the AI (99.1% vs. 95.8%) [15]. It is not uncommon that the performance of a single AI algorithm varies in different clinical settings [18]. Their scanning protocol was fairly similar to our scanning protocol, but the reference standard was obtained using one radiologist with access to the initial report and the AI output who, in case of doubt, could request the judgement of another senior radiologist. The difference in sample size and prevalence of PE may also at least in part explain the discrepancies between our study and the study of Cheikh et al.

Our results might be suggestive of a general statement that AI could replace radiologists as it reached higher diagnostic accuracy than the initial report of the attending radiologist. This has for example been previously suggested for digital mammography screening [20].

However, standalone use of AI algorithms in reading CTPA is currently not warranted as reading CTPAs doesn't solely include analysis of possible occlusion of the pulmonary arteries. Radiologists can identify other pathology and thus remain responsible for good patient care [21]. Aside from the possibility of the AI missing clinically relevant PE without the additional reading of the radiologist, current algorithms focused on the detection of PE will miss other relevant findings. This might include thrombus in the right atrial appendage, signs of significant pulmonary hypertension and right-ventricular pressure overload, and a variety of additional clinically relevant findings of infectious, oncological or cardiovascular aetiology [22–25].

A strength of our study was the use of a large consecutive cohort of > 3000 patients in an academic medical centre with a PE prevalence of 21.6%, reflecting every day clinical practice [26, 27]. As the AI found 60 additional cases of PE in our study population of 3316 patients, to detect a single additional acute PE case would require a number-needed-to-analyse by AI of 56 patients. Due to the low rate of false positive AI findings, evaluating the AI output could be considered minimal additional effort for radiologists.

### Limitations

This study comes with some limitations. We did not re-evaluate all images of patients that were classified positive by both the radiology report and the AI output and negative by both the radiology report and AI algorithm. It has been observed that for small PE there can be substantial disagreement between readers depending on expertise level and that small PE are often overdiagnosed in routine practice [28]. However, missed or false positive cases by both would not have affected the outcome of the comparison between the radiology report and the AI output.

The retrospective nature of our study did not allow a direct comparison of conventional care and AI-assisted care, which is considered the ideal design to evaluate the additional value of AI [29]. This would require a preferably prospective study design with large paired or parallel groups in a hospital where AI is implemented. However, the seamless workflow integration of the algorithm complicates the conduct of such a prospective clinical study design, since the AI output is readily available to the radiologist. Another possibility would be to compare diagnostic accuracy of radiologists before and after the implementation of AI, which has the limitation of the time interval between the evaluation before and after implementation. Moreover, to acquire an acceptable reference standard for such a design, several radiologists would have to do a consensus reading of a large number of scans. It could be argued whether such a large investment in effort and costs is still required with the current excellent diagnostic accuracy results of this AI in mind.

### Future perspectives

Our results combined with those of previous studies call out for clinical utility studies to determine the cost-effectiveness and the impact of AI assistance on healthcare quality and efficiency, as a next step towards reimbursement and clinical adoption of AI assistance for the detection of PE in CTPA [30–32].

## Conclusion

In conclusion, this study showed that the AI algorithm had a significantly higher diagnostic accuracy to detect PE on CTPA of patients suspected of PE compared to the report of the attending radiologist. This indicates that missed positive findings can be prevented with the implementation of AI-assisted care in daily clinical practice.

## Data Availability

The datasets used and/or analysed during the current study are available from the corresponding author on reasonable request.

## Abbreviations

AI: Artificial intelligence
CI: Confidence interval
CTPA: Computed tomography pulmonary angiography
PE: Pulmonary embolism
